# Supplementary figures and images for: Image-based phenotyping of cassava roots for diversity studies and carotenoids prediction
Source: PLoS One. 2022 Jan 31;17(1):e0263326. doi: 10.1371/journal.pone.0263326 (PMC8803208; doi:10.1371/journal.pone.0263326)

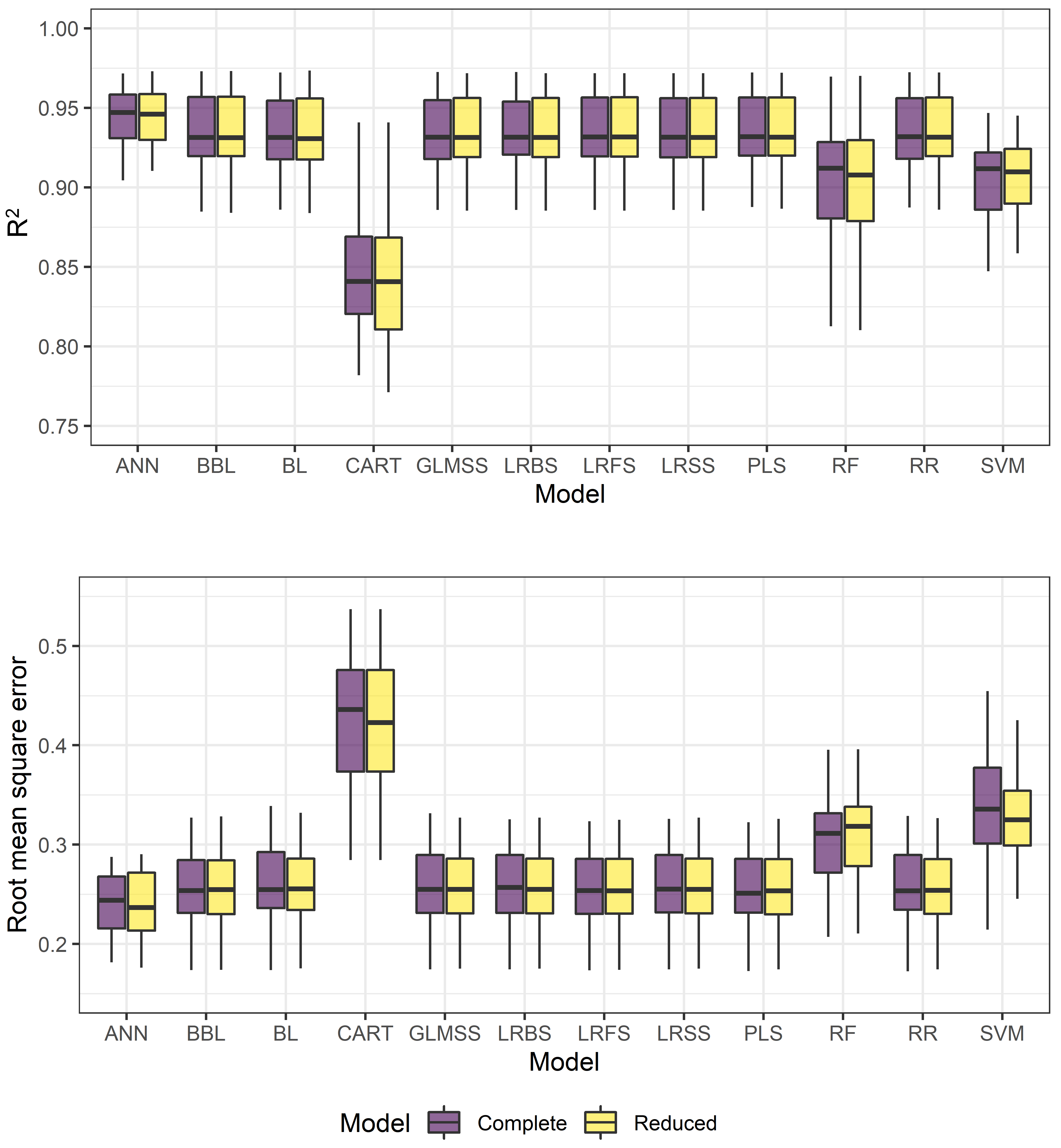

Supplement: S1 Fig — Artificial Neural Network (ANN), Bayesian Blasso (BBL), Bayesian Lasso (BL), Classification and Regression Trees (CART), Generalized Linear Model with Stepwise Feature Selection (GLMSS), Linear Regression with Backward Selection (LRBS), Linear Regression with Forward Selection (LRFS), Linear Regression with Stepwise Selection (LRSS), Partial Least Squares (PLS), Random Forest (RF), Ridge Regression (RR), and Support Vector Machine (SVM). (TIFF) [file pone.0263326.s003.tiff]

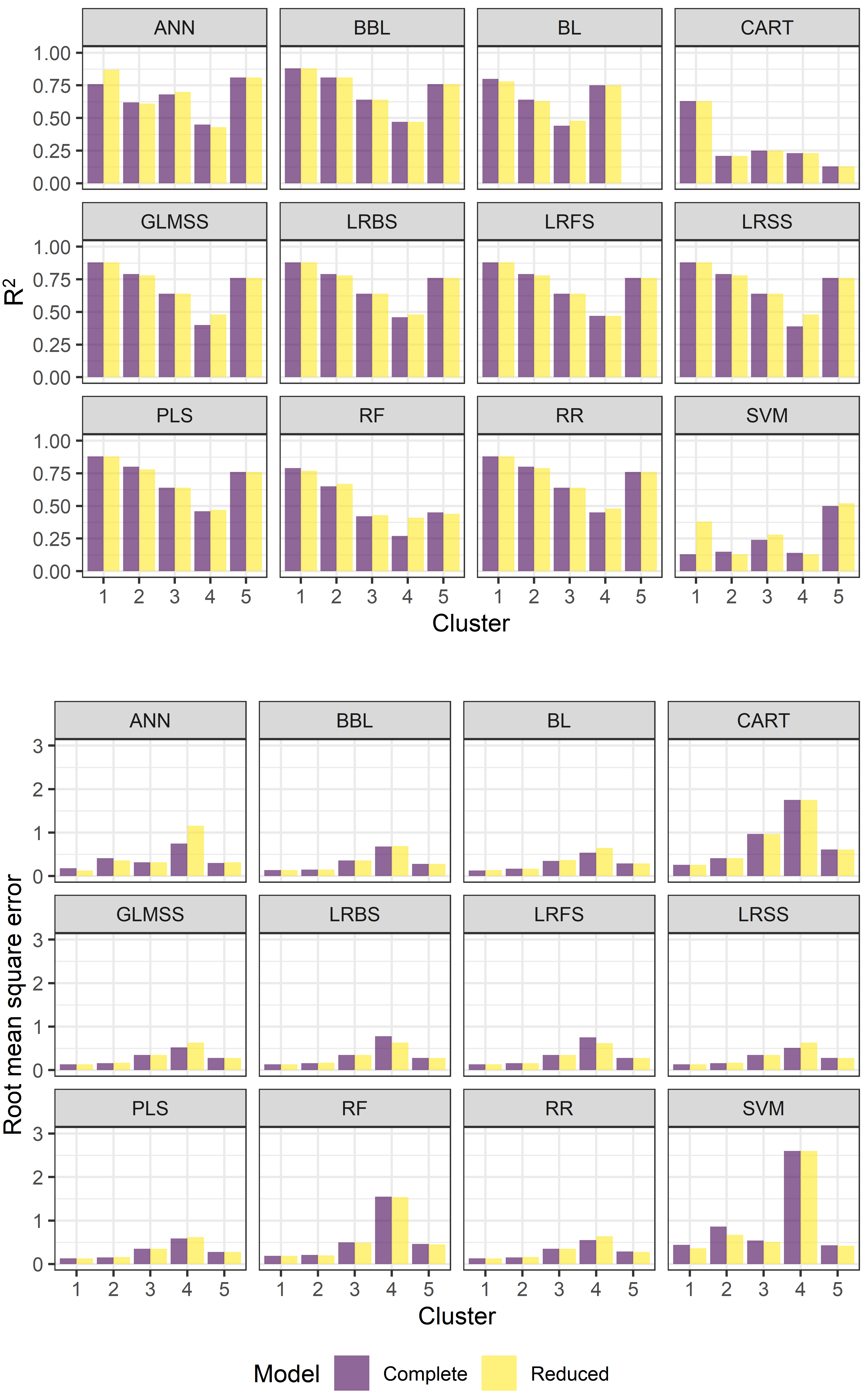

Supplement: S2 Fig — Artificial Neural Network (ANN), Bayesian Blasso (BBL), Bayesian Lasso (BL), Classification and Regression Trees (CART), Generalized Linear Model with Stepwise Feature Selection (GLMSS), Linear Regression with Backward Selection (LRBS), Linear Regression with Forward Selection (LRFS), Linear Regression with Stepwise Selection (LRSS), Partial Least Squares (PLS), Random Forest (RF), Ridge Regression (RR), and Support Vector Machine (SVM). (TIFF) [file pone.0263326.s004.tiff]

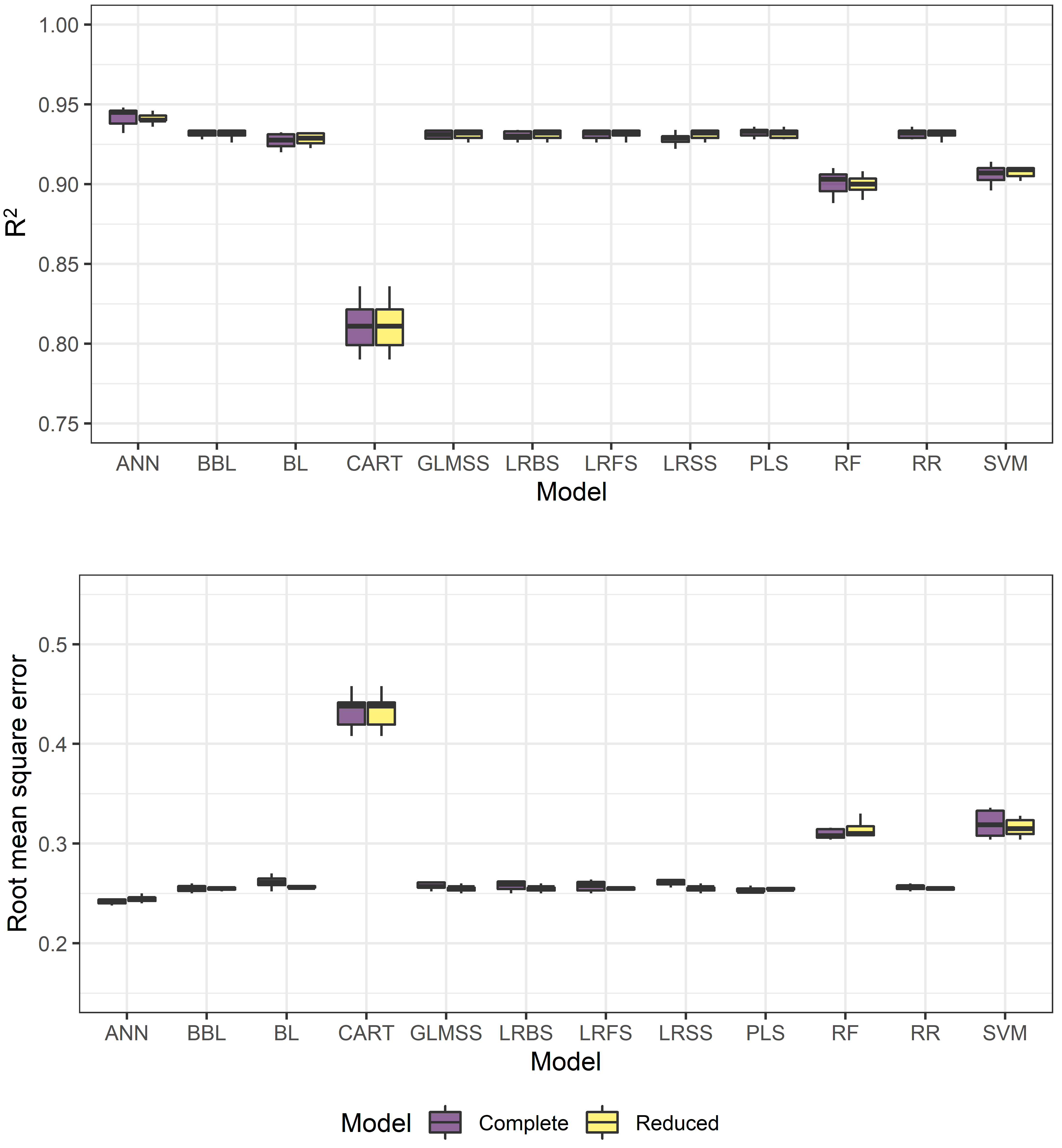

Supplement: S3 Fig — Artificial Neural Network (ANN), Bayesian Blasso (BBL), Bayesian Lasso (BL), Classification and Regression Trees (CART), Generalized Linear Model with Stepwise Feature Selection (GLMSS), Linear Regression with Backward Selection (LRBS), Linear Regression with Forward Selection (LRFS), Linear Regression with Stepwise Selection (LRSS), Partial Least Squares (PLS), Random Forest (RF), Ridge Regression (RR), and Support Vector Machine (SVM). (TIFF) [file pone.0263326.s005.tiff]
