# Supplementary material for: Image-based phenotyping of cassava roots for diversity studies and carotenoids prediction
Source: PLoS One. 2022 Jan 31;17(1):e0263326. doi: 10.1371/journal.pone.0263326 (PMC8803208; doi:10.1371/journal.pone.0263326)
Supplement: Attachment — Submitted filename: Carvalho et al_Plos One_2021 Response to Reviewers.docx [file pone.0263326.s006.docx]

**Reviewer #1:**

1. From line 31 to line 34, using two sentences to describe the methods of data analysis and prediction models is cumbersome, and you can combine these two sentences.

**Response:** There was an error here. The two sentences were duplicated. Thank you.

2. In line 121, select the standard root for image collection. What is the standard root defined?

**Response:** We clarified this sentence:

“During harvest, 4–6 commercial roots (>5 cm in diameter and around 12–20 cm in length) of all 228 genotypes were selected.”

3. In Fig 1., how to understand the shaded area next to the blue line?

**Response:** It is the 95% confidence region. We changed the caption of Figure 1.

4. In Fig 5., ordinate of each figure use “L., a., b.” which is not accurate, it should be in line with “L*, a*, b*”. What’s more, abscissa of each figure should be added with units (%), or it should be explained in the notes of the figure.

**Response:** We changed the figures according to reviewer’s suggestion.

5. In Table 1., the column of “Modelo” might be “Model”.

**Response:** Ok, thanks.

6. In line 265, “performed similarly (RMSE = 0.23, R2 = 0.93)” should be “RMSE = 0.26”.

**Response:** Ok, thanks.

7. From line 265 to 267, “…had high predictive abilities (R2 = 0.91 and 0.90, respectively)”, the values of R2 come from different model (Complete model and Reduced model, respectively). But “they also had higher RMSE values (0.32 and 0.33, respectively)”, the values of RMSE come from the same model (Reduced model). How should we understand it here?

**Response:** We tried to clarify the sentence as:

“Although the RF and SVM models had high predictive abilities (R2 = 0.90 for both complete and reduced models), they also had higher RMSE values (0.31 and 0.32 for complete and reduced model, respectively (RF) and 0.33 for both complete and reduced (SVM).”

8. In Fig 6. and its notes, there is no description of total carotenoid content units.

**Response:** We added the unit (ug.g^-1^). Thanks.

9. What is the significance of the simplified model? Compared with the complete model, the prediction effect of simplified model is not significantly improved, and the prediction ability of some models is even decreased.

**Response:** The paired t-test was performed based on repeated k-folds cross-validation to compare the prediction algorithms of the complete and reduced models.

10. Is the root that was ground into powder the same sample as the root that collected image information? If it is, during the process from root to powder (including the image acquisition process), the root is exposed to air, is the TCC content stable?

**Response:** The roots used for capturing imagens were the same grounded for the TCC analysis. Although the carotenoids are sensitive to ultraviolet light and high temperature, we adopted different strategies to minimize any adverse changes in TCC due to such effects protecting them from UV light (by covering the samples with aluminum foil to avoid contact with light, whenever possible) and avoiding excessively high temperatures using environment with controlled temperature. Please see the changes in Material and methods.

11. The MAPE of each model is suggested to add in the main content.

**Response:** We added the mean absolute percentage error

**Reviewer #2:**

This study used RGB images of cassava roots to predict total carotenoids contents with a 228 genotypes population from cassava germplasm. Chromatic components L*, a* and b* as well as hue and lightness were calculated from the RGB values with the CIELAB system. As the correlation between each of these parameters, eg. L*, a*, b*, hue and lightness with total carotenoids contents is not very high, so the author tried to develop models based on these parameters (eg. L*, a*, b*, hue and lightness) to predict the total carotenoids contents and got very high correlation coefficients. Especially, this study used 12 different models.
However, I have two major concerns.

First, I did not find the model validation with independent dataset. I suggest the author used part of the data to build model and use the other part of the data to validate the models.

Second, how the PCA analysis and k-means clustering necessary or useful in logic to developing the prediction models? I see that the 228 genotypes can be perfectly clustered into 5 clusters by the phenotypes, so I suggest to use different clusters to develop models and use the left clusters to validate. I assume that the models developed based on different clusters should have different predictivity.

**Response:** We added three cross-validation approaches to get the accuracy, MAPE and RMSE parameters. Please see in “Material and methods” and “Results” sections.

The methods and equations for calculating L*, a* and b* were not presented in the Methods.

**Response:** The equations for converting RGB values into CieLab values were included in the manuscript

L137-143, the equations should be presented in separate rows, not mixed in text. This should be applied through the manuscript.

**Response:** Ok!

Fig1 legend, the blue line is not for 1:1.

**Response:** There was an error here. The blue line represents the regression line.

Fig6 legend, the blue line is not for 1:1 isoline from the figure.

**Response:** The same error of Figure 1. We have changed. Thanks.
